# The Coping Circumplex Model: An Integrative Model of the Structure of Coping With Stress

**DOI:** 10.3389/fpsyg.2019.00694

**Published:** 2019-04-16

**Authors:** Krzysztof Stanisławski

**Affiliations:** Institute of Psychology, Cardinal Stefan Wyszyński University in Warsaw, Warsaw, Poland

**Keywords:** coping style, coping strategy, coping mode, stress, structure of coping, Coping Circumplex Model, problem coping, emotion coping

## Abstract

It seems obvious that the identification of coping structure is necessary to understand how stress affects human health and functioning. Despite numerous coping conceptualization proposals, there is no agreement as to the core coping categories. This article presents the Coping Circumplex Model (CCM), which is designed to integrate various coping distinctions, drawing inspiration from the tradition of circumplex models in psychology. The model is based on the assumption that individuals in stressful situations face two tasks: they need to solve the problem and regulate their emotions, which are reflected in two corresponding dimensions, that is, the problem coping dimension and emotion coping dimension. Problem coping and emotion coping are interpreted as bipolar dimensions. Importantly, these dimensions define a space for other coping categories. The model contains a total of eight coping styles forming a circumplex: positive emotional coping, efficiency, problem solving, preoccupation with the problem, negative emotional coping, helplessness, problem avoidance, and hedonic disengagement. The paper discusses the potential of the CCM to overcome some of the problems of stress psychology by: (a) supplementing the set of coping categories (i.e., process, strategy, style) with coping mode; (b) providing a foundation for the integration of numerous coping constructs; (c) enabling the interpretation of results obtained by means of different coping measures, thus facilitating knowledge consolidation; (d) explaining relationships between coping and adjustment after trauma, as well as explaining the mechanisms of psychological interventions (e.g., cognitive therapy, exposure therapy); (e) clarifying linkages between the effectiveness of coping strategies and situation controllability. Moreover, the CCM may elucidate the relationship between coping and emotion regulation (e.g., cognitive reappraisal and expressive suppression).

## Introduction

An understanding of coping structure is crucial to explaining the impact of stress on physical and mental health and well-being. However, there is little agreement as to the structure of coping, with at least 100 coping taxonomies and 400 lower-order categories proposed in the literature (Skinner et al., 2003). Therefore, the possibility of application of the same coping categories across different questionnaires is very limited, which impedes the consolidation of findings from various studies (cf. Compas et al., 2001; Skinner et al., 2003). Furthermore, it appears that an understanding of the relationships between external variables (e.g., mental health) and coping is incomplete (e.g., Carver et al., 1989; Scheier et al., 1994). Similarly, the mechanisms of interventions aimed at improving coping are still unclear (Coyne and Racioppo, 2000) and effectiveness of endorsed coping strategy depending on situational variables requires at least more clarification (cf. Park et al., 2001). Indeed, it seems that a new model of coping structure is needed to resolve the afore-mentioned issues. A proposal of such a model shall be given following a brief discussion of the major approaches to the coping structure (e.g., Lazarus and Folkman, 1984; Carver et al., 1989; Parker and Endler, 1992).

### Review of Selected Coping Models

#### Problem-Focused and Emotion-Focused Coping by Lazarus and Folkman (1984)

Lazarus and Folkman (1984) distinguished two basic coping categories, i.e., problem-focused and emotion-focused coping, as responses aimed at “managing or altering the problem causing the distress” and “regulating emotional responses to the problem,” respectively (Lazarus and Folkman, 1984, p. 150). They developed the Ways of Coping Questionnaire (WCQ) based on the problem- vs. emotion-focused distinction to measure responses to stress during a specified period of time (Folkman and Lazarus, 1980, 1985). Solutions with different numbers of WCQ factors have been extracted, e.g., two (Folkman and Lazarus, 1980), four (Chan, 1994; Van Liew et al., 2016), five (Sørlie and Sexton, 2001), seven (Mishel and Sorenson, 1993), and eight (Folkman and Lazarus, 1985; Folkman et al., 1986a). Folkman et al. (1986a, p. 995) identified the eight factors shown in Table 1.

**Table 1:** Definitions of coping strategies from the WCQ.

**Coping strategy**	**Definition**
Planful problem-solving	“deliberate problem-focused efforts to alter the situation”
Escape–avoidance	“wishful thinking and behavioral efforts to escape or avoid”
Accepting responsibility	“acknowledges one's own role in the problem with a concomitant theme of trying to put things right”
Positive reappraisal	“create positive meaning by focusing on personal growth”
Confrontive coping	“aggressive efforts to alter the situation”
Distancing	“efforts to detach oneself” and “creating a positive outlook”
Self-controlling	“regulate one's own feelings and actions”
Seeking social support	“seek informational support and emotional support”

*All definitions are derived from Folkman et al. (1986a, p. 995)*.

Lazarus and Folkman have made an important step in the conceptualization of coping, searching its structure and influenced on the formation of many other coping models (e.g., Carver et al., 1989; Tobin et al., 1989; Endler and Parker, 1990). However, the distinction between problem- and emotion-focused coping has been criticized by many authors over the years (Lazarus, 1996; Compas et al., 2001; Skinner et al., 2003). Later on in his career, Lazarus (1996) admitted that the distinction between problem- vs. emotion-focused coping “led to an oversimple conception of the way coping works” (p. 292). [Skinner et al. (2003), p. 227] stated that “most ways of coping can serve both functions and thus could fit into both categories. For example, making a plan not only guides problem solving but also calms emotion. Venting not only escalates negative emotion but also interferes with implementing instrumental actions.” In a similar vein, Compas et al. (2001) noted that a single coping strategy may be focused both on the problem and emotions. Problem-focused vs. emotion-focused coping is not conceptually clear (Skinner et al., 2003); particularly problematic is emotion-focused coping, which is composed of very diverse coping categories (Compas et al., 2001). For instance, in some taxonomies the tendency to calm oneself (e.g., “not worrying”) is classified as emotion-focused coping (Tolor and Fehon, 1987), whereas in other models emotional discharge is regarded as such. Furthermore, this distinction is not exhaustive (cf. Band and Weisz, 1988; Carver et al., 1989) as it leaves out social support seeking (Skinner et al., 2003).

#### Coping Dimensions Derived Theoretically by Carver et al. (1989)

Carver et al. (1989) worked from the assumption that the distinction between problem-focused and emotion-focused coping is useful, but insufficient (Carver et al., 1989). Based on the literature, they identified 13 dimensions of coping: five interpreted as sub-dimensions of problem-focused coping (i.e., active coping, planning, suppression of competing activities, restraint coping, seeking social support for instrumental reasons), and another five as sub-dimensions of emotion-focused coping (seeking social support for emotional reasons, positive reinterpretation and growth, acceptance, denial, turning to religion); the remaining three were classified “less useful” strategies (focus on and venting of emotions, behavioral disengagement, mental disengagement) (Carver et al., 1989). To measure these 13 coping strategies Carver et al. (1989) developed the COPE inventory, which has now been extended to include two additional scales: humor and substance use. The definitions of all scales are shown in Table 2.

**Table 2:** Definitions of coping strategies from the COPE.

**Coping category**	**Definition**
**PROBLEM-FOCUSED COPING**	
Active coping	“process of taking active steps to try to remove or circumvent the stressor or to ameliorate its effects. Active coping includes initiating direct action, increasing one's efforts, and trying to execute a coping attempt in stepwise fashion”
Planning	“thinking about how to cope with a stressor. Planning involves coming up with action strategies, thinking about what steps to take and how best to handle the problem”
Suppression of competing activities	“putting other projects aside, trying to avoid becoming distracted by other events, even letting other things slide, if necessary, in order to deal with the stressor”
Restraint coping	“waiting until an appropriate opportunity to act presents itself, holding oneself back, and not acting prematurely”
Seeking social support for instrumental reasons	“seeking advice, assistance, or information”
**EMOTION-FOCUSED COPING**	
Seeking social support for emotional reasons	“getting moral support, sympathy, or understanding”
Positive reinterpretation and growth	“construing a stressful transaction in positive terms”
Acceptance	Learning to accept the reality of a stressful situation*
Denial	“reports of refusal to believe that the stressor exists or of trying to act as though the stressor is not real”
Turning to religion	“tendency to turn to religion in times of stress”
**“LESS USEFUL”**	
Focus on and venting of emotions	“the tendency to focus on whatever distress or upset one is experiencing and to ventilate those feelings”
Behavioral disengagement	“reducing one's effort to deal with the stressor, even giving up the attempt to attain goals with which the stressor is interfering”
Mental disengagement	“wide variety of activities that serve to distract the person from thinking about the behavioral dimension or goal with which the stressor is interfering,” e.g., daydreaming, watching TV, escaping through sleep
**TWO ADDITIONAL SCALES**	
Humor	Dealing with negative emotions through humor*
Substance use	Use of alcohol or drugs to disengage from a stressor or feel better*

Definitions were taken from Carver et al. (1989, pp. 268–270) or were developed based on items (^*^).

In general, the distinction between problem-focused and emotion-focused coping used by Carver et al. (1989) was not confirmed by analysis of the higher-order structure of the COPE. Furthermore, exploratory analyses of the COPE scales have demonstrated solutions with different numbers of factors: three (e.g., Stowell et al., 2001), four (Carver et al., 1989), or five (Deisinger et al., 1996; Sica et al., 1997). In turn, the greatest advantages seem to be a wide range of coping strategies, the existence of two versions of the questionnaire (dispositional and situational), and utility proven in many studies (e.g., Fontaine et al., 1993; Scheier et al., 1994; Maltby and Day, 2000; Sonnentag and Fritz, 2007).

#### Task-Oriented, Emotion-Oriented, and Avoidance-Oriented Coping by Parker and Endler (1992)

Parker and Endler (1992) observed that many coping measures are characterized by methodological shortcomings which preclude generalization of results from one population to another. Their goal was to change this situation by introducing a new instrument based on three coping styles. The first two referred to problem- vs. emotion-focused coping (Lazarus and Folkman, 1984). Parker and Endler (1992) noted that problem-focused coping strategies are associated with a task-orientation, whereas emotion-focused ones reflect a person-orientation: “task-orientation refers to strategies used to solve a problem, reconceptualize it (cognitively), or minimize its effects” (Parker and Endler, 1992, p. 325) and “person-orientation refers to strategies that may include emotional responses, self-preoccupation, and fantasizing reactions” (Parker and Endler, 1992, p. 325). According to Parker and Endler (1992), many coping models distinguished a third basic dimension–avoidance-oriented coping, involving both task-oriented, and person-oriented strategies. Task-oriented avoidance is conceptualized as distraction, while person-oriented avoidance takes the form of social diversion. A person may avoid a stressful situation by engaging in substitute activities (distraction–e.g., watching TV) or seeking out other people (social diversion). “In task-oriented coping, the person is confronting the stressful task. In distraction coping, the person is substituting an alternative task of his or her choosing” (Parker and Endler, 1992, p. 326). On the other hand, social diversion is “person-oriented in that the individual tries to “lose himself or herself” by being with other persons rather than confronting the stressful situational task” (p. 326). To measure the three coping styles, Endler and Parker (1999) developed the Coping Inventory for Stressful Situations (CISS).

In contrast to most coping inventories, the CISS has revealed satisfactory psychometric properties and a stable factor structure confirmed across different cultures (e.g., Furukawa et al., 1993; Strelau et al., 2005; Rafnsson et al., 2006; Boysan, 2012). The most significant limitation of the model is that it encompasses only three coping categories and cannot explain a plethora of coping responses (cf. Schwarzer and Schwarzer, 1996).

### Coping Research Problems

Problems associated with coping research include a lack of agreement as to the structure of coping (Compas et al., 2001; Skinner et al., 2003), prediction of external variables using the coping construct, difficulties in measuring emotional coping responses (Stanton et al., 1994; Compas et al., 2001), mechanisms of interventions aimed at improving coping (Coyne and Racioppo, 2000) and effectiveness of coping depended on situational conditions (Lazarus and Folkman, 1984; Park et al., 2001). First, the serious problem is lack of consensus about coping structure (Compas et al., 2001; Skinner et al., 2003). Authors pointed that coping measures have revealed problems with replication of structure across samples (Schwarzer and Schwarzer, 1996; Compas et al., 2001; Skinner et al., 2003; Sveinbjornsdottir and Thorsteinsson, 2008) and it is difficult to apply the same coping categories across different questionnaires and samples (cf. Compas et al., 2001; Skinner et al., 2003). A detailed comparison of coping scales is needed to determine whether the findings obtained thereby can be aggregated. A lack of agreement on core coping categories hinders the consolidation of knowledge (Skinner et al., 2003).

Second, it is often reported that some external variables (e.g., mental health indicators) are quite consistently related to the configuration of specific constructs adopted by a given model. However, those combinations are not directly predicted by the theoretical models. For example, in some studies distress was associated with a configuration of three coping categories from the CISS (Endler and Parker, 1999): low task-oriented, high emotion-oriented, and high distraction, with this particular combination labeled as maladaptive coping (Dunkley and Blankstein, 2000). It is worth noting that in this research the latent variable of maladaptive coping exhibited a strong loading only for emotion-oriented coping (Dunkley and Blankstein, 2000). In the study of Leandro and Castillo (2010), a similar set of CISS constructs was associated with trait anxiety and depression (instead of distraction, avoidance-oriented coping). Other studies have indicated a positive correlation of anxiety with emotion-oriented coping (McWilliams et al., 2003; Cohan et al., 2006) and distraction as well as a negative correlation with task-oriented coping (Cohan et al., 2006). Depression has been found to be associated with emotion-oriented coping (McWilliams et al., 2003; Cohan et al., 2006) and inversely with task-oriented coping (Cohan et al., 2006). In agreement with Dunkley and Blankstein (2000), other authors have noted that among the CISS styles, emotion-oriented coping reveals the strongest correlation with distress (McWilliams et al., 2003; Cohan et al., 2006; Leandro and Castillo, 2010). The configuration of three CISS variables predicting distress cannot be derived simply from the Endler and Parker model (1999), which suggests the existence of a new coping category.

Likewise, in some other measures one external variable is associated with more than one coping category. Self-esteem is correlated with at least five of the 13 strategies included in the COPE (Carver et al., 1989; Scheier et al., 1994). Self-esteem has been found to be positively associated with active coping, planning, positive reinterpretation and growth, and negatively with denial and behavioral disengagement (Carver et al., 1989; Scheier et al., 1994). Active coping and planning have been classified as problem-focused coping (O'Connor and O'Connor, 2003; Litman, 2006), task coping (Kallasmaa and Pulver, 2000) or the active factor/ rational coping (Stowell et al., 2001), whereas denial and behavioral disengagement has been associated with avoidant coping (Kallasmaa and Pulver, 2000; Stowell et al., 2001; O'Connor and O'Connor, 2003; Litman, 2006). Positive reinterpretation and growth has been assigned to cognitive reconstruction (O'Connor and O'Connor, 2003) or the active factor/ rational coping (Lyne and Roger, 2000; Stowell et al., 2001).

It is not known why self-esteem is correlated with the aforementioned coping strategies. In addition, different authors may assign the same coping strategies to different higher-order categories (Carver et al., 1989; Kallasmaa and Pulver, 2000; O'Connor and O'Connor, 2003; Litman, 2006). Indeed, the relationship between self-esteem and the COPE strategies as well as between distress and the CISS scales cannot be easily explained within the existing basic coping categories. Currently, it seems impossible to elucidate the general mechanism of associations between coping and external variables (e.g., self-esteem) and to test it irrespective of the instruments used.

Third, some authors have pointed to the need of including a broader spectrum of positive emotional regulation in coping inventories (Stanton et al., 1994; Compas et al., 2001). This seems particularly interesting in conjunction with the fact that while coping and emotion regulation are distinct categories, they reveal meaningful commonalities (Compas et al., 2017). Cognitive reappraisal, expressive suppression (Gross and John, 2003), distraction, social control, worry, and punishment (Wells and Davies, 1994) are instances of emotion regulation strategies. A comprehensive model of coping structure should enable the integration of coping with other types of constructs, e.g., emotion regulation.

Fourth, there is a gap between coping theory and practice (Coyne and Racioppo, 2000). While coping skills interventions seem to be effective (e.g., Steenkamp et al., 2015), the foundations on which they are based are yet to be elucidated. There is limited knowledge about the essential elements of intervention-facilitated change and barriers to development (Coyne and Racioppo, 2000).

The final problem concerns the effectiveness of particular coping strategies with regard to situational variables, e.g., controllability (cf. Lazarus and Folkman, 1984). While in some studies problem-focused coping was positively related to distress in uncontrollable situations (e.g., Terry and Hynes, 1998), in other investigations it was negatively associated with distress under similar conditions (e.g., Taylor et al., 2008). These contradictory findings cannot be reconciled under the current paradigm. Thus, a comprehensive model integrating the structure of coping with stress is needed to overcome at least some of the aforementioned issues.

## The Coping Circumplex—Theoretical Proposal

### Basic Assumptions

#### Defining Coping

Coping can be conceptualized as intentional and conscious responses to stress (e.g., Compas et al., 2001), intentional, conscious or unconscious responses to stress (e.g., Lazarus and Folkman, 1984), and as both intentional or automatized responses to stress (e.g., Skinner and Wellborn, 1994).

The presented theoretical model assumes that coping refers to both volitional and automatized, cognitive, emotional, and behavioral responses to stress. This definition is very similar to the conceptualization of Skinner and Wellborn (1994), but it is free from the context of a particular model (i.e., theory of needs). Therefore, it is more universal and more appropriate for a general model of coping structure.

There are three arguments for a definition of coping to include both volitional and automatized responses to stress:

1. It is difficult to decide unequivocally whether a given response to stress is conscious or automatized (Coyne and Gottlieb, 1996; Snyder and Pulvers, 2001; Skinner et al., 2003). According to Skinner et al. (2003), one stress response can be more or less conscious or automatized under different situations. Moreover, deliberative behavior can become more automatized during repetition (Snyder and Pulvers, 2001).
2. It is problematic or impossible to determine which items comprising the coping measures refer to deliberate or automatized responses to stress (Coyne and Gottlieb, 1996); examples include: “I sleep more than usual” from the COPE (Carver et al., 1989) and “Become very upset” from the CISS (Endler and Parker, 1999).
3. The removal of involuntary responses from coping research would place those variables in the area of unexplained variation, which would impede a more comprehensive account of the relationship between coping and its outcomes (cf. Coyne and Gottlieb, 1996).

#### The Problem Coping and Emotion Coping

In stressful situations, individuals face two tasks: they need to solve the problem and regulate their emotions (cf. Lazarus and Folkman, 1984). The two tasks correspond to two dimensions: problem coping (describing whether the person solves the problem or avoids the problem) and emotion coping (describing how the individual regulates one's emotions under stress). These dimensions are similar to the problem-focused coping and emotion-focused coping (Lazarus and Folkman, 1984), but they also take into account the criticism formulated against Lazarus and Folkman (1984) (e.g., Skinner et al., 2003).

Problem coping and emotion coping may be connected with the two dimensions introduced by Gol and Cook (2004), which define the space within which to locate coping strategies. These dimensions are approach-avoidance and emotional equilibrium-disequilibrium. Approach-oriented coping stands for the cognitive efforts aimed at finding a solution to the problem, understanding its causes and accepting it, while avoidance-oriented coping implies distracting oneself from the stressor. Emotional disequilibrium involves an uncontrolled release of emotions, worries, and suppressing emotions, whereas emotional equilibrium includes strategies of emotion control, relaxation, and calming down (Gol and Cook, 2004).

In reference to the proposal of Gol and Cook (2004), problem and emotion coping may be interpreted as two bipolar dimensions. A high level of problem coping denotes active problem solving (implying active cognitive and behavioral efforts to solve the problem causing the distress), whereas low problem coping indicates problem avoidance (avoiding thinking about the problem and reduced efforts to deal with the stressor). The individual may regulate emotional responses to the problem via positive emotional coping (e.g., using humor or positive reinterpretation) or negative emotional coping (e.g., venting emotions, rumination). High levels of emotion coping are synonymous with positive emotional coping, while the opposite is indicative of negative emotional coping.

The emotion coping dimension reflect the issues articulated in the literature, e.g., “broadening models of stress and coping to include positive as well as negative affect will change the kinds of questions psychologists ask about coping” (Folkman and Moskowitz, 2004, p. 652). Furthermore, emotion coping corresponds to one of two general activation systems of affect, that is, the negative activation dimension (Watson and Tellegen, 1985; Watson et al., 1999). High negative activation refers to fear, hostility, guilt, whereas low negative activation includes serenity and calmness (Watson et al., 1999). Negative and positive emotional coping correspond to high and low levels of negative activation, respectively. Moreover, emotion coping can be related to a combination of two dimensions from the circumplex model of affect: valence and arousal (Russell, 1980; Heller, 1990; Yik et al., 2011). Negative emotion coping is linked to negative valence and high arousal, whereas positive emotion coping is conceptually associated with positive valence and low arousal.

#### Concepts of Coping Strategies and Coping Modes

Coping strategies (or specific coping responses) can be defined as “behaviors, cognitions, and perceptions in which people engage when actually contending with their life problems” (Pearlin and Schooler, 1978, p. 5). However, many authors use the concept of coping strategy without defining it (Band and Weisz, 1988; Carver et al., 1989; Amirkhan, 1990; Worthington and Scherer, 2004; De Boo and Wicherts, 2009), which, given the large number of coping categories, many of which are similar (Skinner et al., 2003), may lead to confusion.

A valuable guidance in developing the concept of coping strategy is the observation of Skinner et al. (2003) that homogeneous coping categories should enable the pursuit of the same goals. Furthermore, it has been emphasized that an understanding of coping goals is necessary to gain an insight into coping acts (Band and Weisz, 1988; Schwarzer and Schwarzer, 1996; Coyne and Racioppo, 2000). It can be assumed that the specific coping goals are carried out by corresponding coping functions, e.g., the goal of problem solving can be implemented by the function of problem solving. The conceptualization of coping strategy proposed by Pearlin and Schooler (1978) is very general and does not include coping functions. In the presented paper, a coping strategy is defined as a cognitive, emotional and/ or behavioral response to stress associated with a particular function, e.g., calming down or solving the problem.

While some strategies may involve very similar responses in terms of processes or behaviors, they can nevertheless fulfill different functions. This variability may be addressed by the notion of coping mode understood as a set of coping strategies, which include very similar cognitive, emotional, and/or behavioral responses to stress, but are associated with different functions. Both coping strategies (Carver et al., 1989; cf. Carver, 1997) and modes can be interpreted as states or dispositions.

Coping strategies representing the same coping mode can be distinguished in terms of the problem and emotion coping dimensions. For instance, Band and Weisz (1988) distinguished between problem-focused aggression (i.e., dealing with problems by aggressive reactions) and emotion-focused aggression (i.e., reducing tension by aggressive behaviors). They also identified other strategies: problem-focused crying (i.e., crying to receive support in resolving the problem) and emotion-focused crying (i.e., crying to release feelings or elicit emotional support from others) (Band and Weisz, 1988). Aggression and crying may be interpreted as two different coping modes. It is thought that emotion-focused aggression and emotion-focused crying are examples of negative emotional coping strategies, while problem-focused aggression and problem-focused crying are strategies which reflecting both problem solving and negative emotional coping.

The interpretation of humor from the COPE (Carver et al., 1989) as coping mode may be used to reconcile opposing points of view on this construct. Some authors incorporate humor in one adaptive category with problem-focused strategies (e.g., planning, active coping) (Kallasmaa and Pulver, 2000), while others (Deisinger et al., 1996) include it in hedonistic escapism, a construct consisting of humor and substance use. Some items of the humor scale (Carver et al., 1989) reflect the tendency to calm down (e.g., “I make jokes about it“), whereas others express calming down in conjunction with disregard of the problem (e.g., “I make fun of the situation”). The first can be regarded as a positive emotional coping strategy, while the latter strategy of humor can impede problem resolution by combining problem avoidance with positive emotional coping.

The next example of coping mode is reinterpretation or reappraisal. In their work on religious coping, Pargament et al. (2000) made a distinction between benevolent religious reappraisal (i.e., reformulating the situation as an opportunity for spiritual growth) and punishing God reappraisal (i.e., reinterpreting the stressor as God's punishment). The first strategy corresponds to problem solving and positive emotional coping, whereas the latter one to problem avoidance and negative emotional coping.

Positive reinterpretation or reappraisal has been incorporated in many coping measures (Folkman et al., 1986a; Carver et al., 1989; Ebata and Moos, 1991; Coleman, 1992). The COPE includes a scale of positive reinterpretation and growth with items such as “I look for something good in what is happening” (positive reinterpretation) and “I learn something from the experience” (growth) (Carver et al., 1989). Interestingly, Fontaine et al. (1993), who conducted principal component analysis (PCA) of the COPE items, demonstrated that growth and positive reinterpretation formed two separate factors. Furthermore, growth was correlated with perceived control over stress, while positive reinterpretation was associated with optimism. Thus, COPE growth indicates problem solving and positive emotional coping, whereas COPE positive reinterpretation reflects positive emotional coping. It seems that positive reinterpretation and growth (Carver et al., 1989) is associated with problem solving and positive emotional coping.

The existing distinction of coping categories, i.e., process-strategy-style, has played an important role in stress psychology, but it seems insufficient. The introduction of a fourth category of coping, i.e., a coping mode, which can be considered equally with a process, strategy and style, enables to organize the coping constructs.

#### Definition of Coping Styles

According to Carver et al. (1989), coping strategies may coexist to form a coping style understood as a “preferred set of coping strategies that remains relatively fixed across time and circumstances” (Carver et al., 1989, p. 270). Similarly, Endler and Parker (1999) define coping styles as cognitive/behavioral modes typically used by an individual in various stressful situations.

In search of a new definition of coping styles, one notion seems to be of special significance. Skinner et al. (2003) proposed some criteria for the homogeneity and distinctiveness of coping categories, which is “the extent to which different ways of coping are equifinal, that is, lead to the same goals.…Ways of coping that are functionally homogeneous should be able to be substituted for each other” (p. 247). An integrative perspective can be taken to explore coping styles in greater detail. Coping strategies representing different coping modes can coexist together, creating a coping style. Thus, a coping style is a set of coping strategies which fulfills a specific function and is relatively stable over time as well as across a range of circumstances. The definitions of all coping categories (i.e., style, mode, strategy, and process) are shown in Table 3.

**Table 3:** Coping categories and their definitions.

**Coping category**	**Definition**
Process	“a sequence of strategies changing over time, related to the changes in the characteristics of the situation and changes in the psychophysical state of the individual” (Wrześniewski, 2000, p. 47)
Strategy	Cognitive, emotional and/or behavioral response to stress associated with a particular function, e.g., calming down or solving the problem
Mode	Set of coping strategies, which include very similar cognitive, emotional and/or behavioral responses to stress, but are associated with different functions
Style	Set of coping strategies which fulfills a specific function and is relatively stable over time as well as across a range of circumstances

Similarly to other conceptualizations (Carver et al., 1989; Endler and Parker, 1999), the coping style defined above has broad content and is relatively stable across different situations. As in the proposal of Carver et al. (1989), a coping style is understood here to consist of a set of coping strategies. It is worth to emphasize that the focus on the coping function is the key element distinguishing the presented definition from other proposals (Carver et al., 1989; Endler and Parker, 1999).

### The Coping Circumplex Model

Schwarzer and Schwarzer (1996), p. 107) noted that “Coping with an adversity includes innumerous ways of dealing with diverse person-environment transactions. Thus, coping does not represent a homogeneous concept. Instead, it is a diffuse umbrella term.” This line of reasoning is reflected in the Coping Circumplex Model (CCM), which is based on the concept of bipolar problem coping and emotion coping dimensions as well as on the idea of a circular continuum of coping styles.

#### The Circumplex Tradition in Psychology

The term “circumplex” was introduced by Guttman (1954), being derived from the expression “circular order of complexity.” A circumplex model should meet the following criteria (Gurtman, 1994): (1) Differences between variables are represented by two and only two dimensions; (2) Each variable equidistant from the center of the circle; (3) All variables are uniformly distributed within the hypothetical circle (they are evenly spaced). Furthermore, all possible rotations of the circumplex represent the construct equally well (Larsen and Diener, 1992; Acton and Revelle, 2004).

Within a circumplex, variables form a circular continuum with an arbitrary number of constructs. According to Wiggins (1979), depending on the required level of precision, it is possible to “slice the circumplex pie” (p. 400) into broad or narrow categories (e.g., 4ths, 8ths, 16ths, etc.). For example, the circumplex of vocational interest may contain six (Holland, 1985) or eight types (Tracey and Rounds, 1996). Similarly, eight (Russell, 1980) and 12 constructs (Yik et al., 2011) were distinguished in the circumplex model of affect. Circumplex models have been applied to describe the structure of emotional states (Russell, 1980; Yik et al., 2011), interpersonal dispositions (Leary, 1957; Wiggins, 1995), Big Five personality traits (Hofstee et al., 1992), personality metatraits (Strus et al., 2014; Strus and Cieciuch, 2017), vocational interests (Tracey and Rounds, 1996), identity formation modes (Cieciuch and Topolewska, 2016; Topolewska and Cieciuch, 2016).

The best-established circumplex model in psychology is the Interpersonal Circumplex (IPC). The space of the IPC is defined by two independent dimensions: status (agency, power, dominance) and love (communion, warmth, friendliness) (Leary, 1957; Foa, 1961; Carson, 1969; Wiggins, 1979; Kiesler, 1983). The IPC has been used to describe interpersonal traits (Leary, 1957; Wiggins, 1995), interpersonal values (Locke, 2000) and interpersonal problems (Alden et al., 1990). The IPC has been applied in clinical research to organize and explore the interpersonal nature of personality disorders (Locke, 2000), binge eating disorder (Brugnera et al., 2018), anorexia (Ambwani et al., 2016). The IPC has been found to be useful in the investigation of proneness to specific stressors (Smith et al., 1998) and the interpersonal content of coping (especially catastrophizing; Lackner and Gurtman, 2004).

#### The Circumplex Organization of Coping Styles

It seems clear that one coping response may serve various functions (Band and Weisz, 1988; Schwarzer and Schwarzer, 1996; Compas et al., 2001; Skinner et al., 2003). The question arises as to whether it would be possible to develop a comprehensive model of coping based on completely independent categories. Indeed, it seems more reasonable to strive for a model explaining relationships between coping categories rather than identify pure and mutually exclusive dimensions. Schwarzer and Schwarzer (1996, p. 114) pointed out that “theoretical cross-linked relationships between scales are not considered” in coping measures involving many factors. In the case of the WCQ, Schwarzer and Schwarzer (1996) noted that:

high number of extracted factors is that they do not appear to be all of the same weight or of the same theoretical level. Some may be closer to a higher-order factor or to a general factor, accounting for a larger amount of variance, whereas others may be rather peripheral. It remains undetermined in what way the eight factors are embedded into the initial dimension of problem-focused and emotion-focused functions. There seems to be no empirical evidence for testing such a hierarchy with confirmatory factor analysis (Schwarzer and Schwarzer, 1996, p. 115).

A solution to the above problems may be provided by the CCM. Within the circumplex, categories are not mutually exclusive, some being related to one dimension, and others to both dimensions. Furthermore, in some circumplexes, variables may differ in terms of the strength of their relationship with the two structural dimensions (e.g., the Abridged Big Five Dimensional Circumplex; Hofstee et al., 1992).

**Figure 1:**
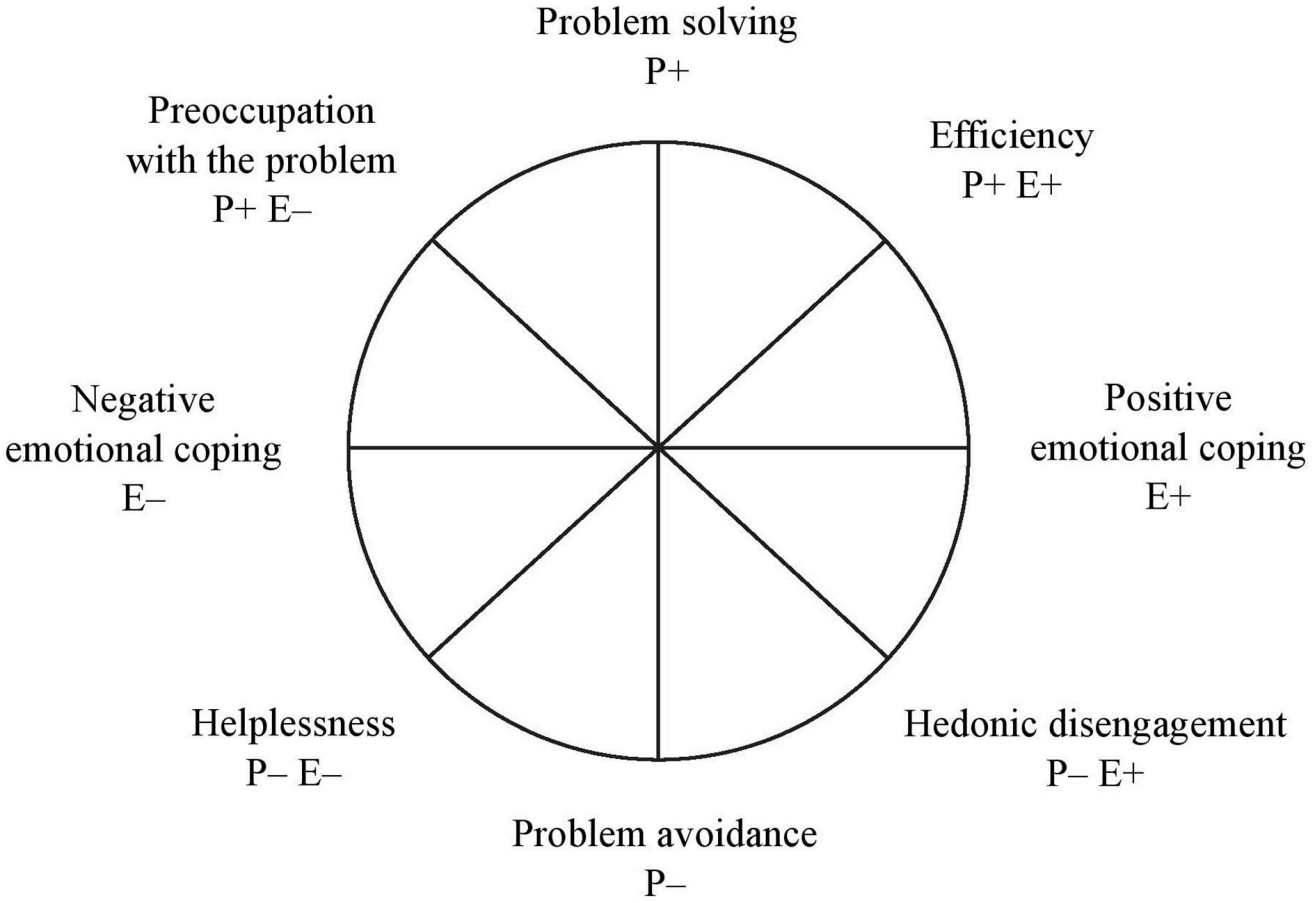
The Coping Circumplex Model.

Assuming that individuals in stressful situations face two tasks–solving the problem and regulating their emotions, one can distinguish two dimensions of coping interpreted as orthogonal axes: problem coping and emotion coping. In the presented model, these dimensions define the space for other coping categories within the circumplex: efficiency vs. helplessness and preoccupation with the problem vs. hedonic disengagement. The CCM contains four bipolar dimensions, which consist of eight coping styles. Each coping style is marked with a symbol derived from the names of the two structuring dimensions. P+ and P– refer to high and low problem coping, while E+ and E– stand for high and low emotion coping, respectively. The following coping styles were identified: problem solving (P+), problem avoidance (P–), positive emotional coping (E+), negative emotional coping (E–), efficiency (P+ E+), helplessness (P– E–), preoccupation with the problem (P+ E–), and hedonic disengagement (P– E+). The CCM is shown in Figure 1.

### The Problem Coping Dimension: Problem Solving vs. Problem Avoidance

#### Definition of Problem Solving

Problem solving involves active cognitive and behavioral efforts to deal with problem. Problem solving consists of acknowledging various thoughts concerning the problem, undertaking efforts to understand the situation, predicting the course of events, choosing the most appropriate solutions, planning to solve the problem and implementing this plan as well as taking consistent action to solve the problem.

Several constructs described in the literature share some common characteristics with problem solving, these are: problem-focused coping (Folkman and Lazarus, 1980, 1985), planful problem-solving (Folkman et al., 1986a), problem solving (Tobin et al., 1989; Amirkhan, 1990), task-oriented coping (Endler and Parker, 1999) as well as active coping and planning (Carver et al., 1989).

Factor analysis of COPE items has revealed that active coping and planning form one factor (Carver et al., 1989; Fontaine et al., 1993). Analysis of the scales indicated that active coping, planning, and suppression of competing activities load the same factor (Carver et al., 1989; Sica et al., 1997), which was labeled “problem solving” by Clark et al. (1995). After analyzing the properties of three coping measures: the COPE (Carver et al., 1989), the Coping Strategy Indicator (CSI, Amirkhan, 1990), and the WCQ (Folkman and Lazarus, 1985), they noted that both COPE and CSI problem solving as well as WCQ problem-focused coping were strongly correlated with each other (Clark et al., 1995). Furthermore, Endler and Parker (1990) found that WCQ problem-focused coping and task-oriented coping from the Multidimensional Coping Inventory (MCI) were significantly correlated. This shows the convergence of the various constructs related to problem solving coping from different inventories.

#### Definition of Problem Avoidance

Problem avoidance consists of the avoidance of thinking about the problem (e.g., by engaging in substitute activities), reducing efforts to solve the problem, postponing task, or giving up attempts to attain goal. As defined above, this coping style exhibits similarities to the constructs of problem avoidance (Tobin et al., 1989), avoidance (Amirkhan, 1990), avoiding problems (Patterson and McCubbin, 1987), escape–avoidance (Folkman et al., 1986a; see Table 4), self-distraction (Carver, 1997), distraction (Endler and Parker, 1999) as well as mental disengagement, behavioral disengagement, and denial (Carver et al., 1989). Using second-order factor analysis, the authors of the COPE demonstrated that both mental and behavioral disengagement as well as denial constitute one factor (Carver et al., 1989), which has been replicated and labeled as “avoidance” (Deisinger et al., 1996; Stowell et al., 2001). It should be noted that the coping constructs connected to problem solving (i.e., active coping and planning) are negatively correlated with problem avoidance strategies (i.e., behavioral disengagement and denial) (Carver et al., 1989).

**Table 4:**
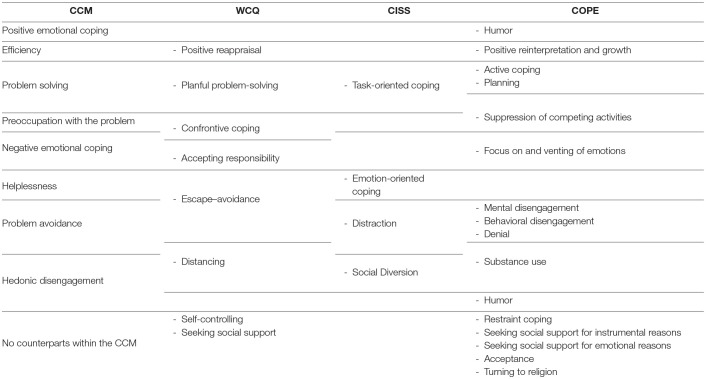
Coping styles from the CCM and corresponding categories from the WCQ, CISS, and COPE.

#### Correlates and Applications of Problem Solving and Problem Avoidance

Desire for control reveals the strongest positive and negative correlations with active problem solving and avoidance strategies, respectively (Gebhardt and Brosschot, 2002). Problem-focused coping is linked to an internal locus of control (Arslan et al., 2009), whereas avoidance to an external locus of control (Lengua and Stormshak, 2000; Scott et al., 2010). Problem-focused coping correlated positively with perceived control over stress and avoidance demonstrated an inverse pattern of relation (Carver et al., 1989; Fontaine et al., 1993).

Task-oriented coping/ problem-focused coping is associated with goal motivation (Struthers et al., 2000; Matthews et al., 2001; Gaudreau et al., 2012) as well as academic goal progress (Struthers et al., 2000; Gaudreau et al., 2012; Saklofske et al., 2012). Problem-focused coping (i.e., active action) is predictive of better job performance (i.e., job attendance and job knowledge), whereas avoidance (i.e., passive adaptation) of inferior performance (i.e., lower quantity and quality of work) (Lu et al., 2010). Among the various COPE strategies, behavioral disengagement and active coping reveal the strongest positive and negative correlations with procrastination, respectively (Sirois and Kitner, 2015).

### The Emotion Coping Dimension: Positive Emotional Coping vs. Negative Emotional Coping

#### Definition of Positive Emotional Coping

Positive emotional coping involves being kind and understanding to oneself as one tries to solve a problem on one's own regardless of success and the use of cognitive transformations that enable the elicitation of positive emotions and calming down (through reinterpretation and humor). It appears that a positive emotional coping shares some characteristics with self-kindness (Neff, 2003), positive reinterpretation (Carver et al., 1989; cf. Fontaine et al., 1993), positive reframing (Carver, 1997), humor (Carver et al., 1989), coping humor (Martin and Lefcourt, 1983), and positive thinking (Connor-Smith et al., 2000).

Self-kindness vs. self-judgment is regarded as one of three components of self-compassion (Neff, 2003). Self-compassion is positively associated with positive automatic thoughts and inversely with negative automatic thoughts (Arimitsu and Hofmann, 2015). Self-compassion is associated with situational positive reinterpretation and growth and reveals a negative correlation with situational focus on and venting of emotions (Neff et al., 2005).

Another construct related to positive emotional coping is humor. As it was mentioned above, humor has at least two strategies, one is similar to positive emotional coping (referring item: “I make jokes about it“; Carver et al., 1989), second to hedonic disengagement. Humor facilitates coping and emotion regulation through positive reinterpretation (Kuiper et al., 1993). Therefore, it is not surprising that based on dispositional version of the Brief COPE, humor and positive reframing (along with acceptance) formed one factor (Dunkley and Blankstein, 2000; Knoll et al., 2005).

Self-kindness, humor and positive reinterpretation have similar correlates (Doron et al., 2014; Brenner et al., 2017). Both self-kindness (Hardin and Larsen, 2014) and humor (Kuiper and Martin, 1993) are inversely associated with actual-ideal self-discrepancies. It was found that self-kindness (Brenner et al., 2017), humor (Kuiper et al., 1993; Doron et al., 2014), and positive reframing (Doron et al., 2014) revealed inverse relationship with perceived stress. Moreover, self-kindness (Hardin and Larsen, 2014), humor (Kuiper et al., 1995), and positive reinterpretation and growth (Schanowitz and Nicassio, 2006) are associated with positive affect, whereas positive reframing is linked to satisfaction at the end of the day (Stoeber and Janssen, 2011). It seems that one latent tendency (i.e., positive emotional coping) is at the root of self-kindness, humor, and positive reinterpretation.

#### Definition of Negative Emotional Coping

Negative emotional coping includes self-criticism when dealing with problem, focusing attention on the negative aspects of stressful situation (e.g., rumination), and on negative emotions (e.g., feelings of tension, pressure, or anger). Negative emotional coping is assumed to be related to self-criticism (Tobin et al., 1989), self-blame (Carver, 1997), accepting responsibility (Folkman et al., 1986a), emotional discharge (Billings and Moos, 1984), venting (Carver, 1997), focus on and venting of emotions (Carver et al., 1989), and rumination (Connor-Smith et al., 2000).

Rumination disposition has been reported to be positively related to self-criticism (O'Connor and Noyce, 2008) and negatively to self-compassion (Raes, 2010). Dispositional focus on and venting of emotions is associated with rumination after stressful events (Cann et al., 2011). Both rumination disposition (Randles et al., 2010) and focus on and venting of emotions (Litman, 2006) are correlated with the behavioral inhibition system (BIS).

Rumination disposition has been shown to be related to the state anxiety (Vǎlenaş et al., 2017), whereas rumination following stressful event to hyperarousal (Cann et al., 2011). Both test and social anxiety are associated with self-criticism (Cunha and Paiva, 2012). Trait anxiety is linked to focus on and venting of emotions (Carver et al., 1989; Dias et al., 2012) as well as self-blame (Dias et al., 2012). Long-term anxiety can be predicted from focus on and venting of emotions (Liverant et al., 2004). More generally, negative affect is associated with the tendency to engage in rumination (Mor and Winquist, 2002; Kvillemo and Bränström, 2014), focus on and venting of emotions (Kato, 2013), venting (Kvillemo and Bränström, 2014) as well as self-blame (Kvillemo and Bränström, 2014). Self-criticism, self-blame, focus on and venting of emotions as well as rumination have very similar correlates. Presumably, one construct (i.e., negative emotional coping) underpins all of the above-mentioned responses.

### Efficiency vs. Helplessness

Some forms of coping focus both on problems and emotions; for example, Lyne and Roger (2000) and Stowell et al. (2001) identified active coping/rational coping – a higher-order dispositional category encompassing both problem-focused coping and positive reinterpretation and growth, acceptance as well as restraint coping. In the present paper, the configuration of problem solving and positive emotional coping is labeled as efficiency. The opposite pole is helplessness, which is a combination of problem avoidance and negative emotional coping.

#### Definition of Efficiency

Efficiency is a combination of problem solving and positive emotional coping. Efficiency involves the acknowledgment of thoughts and feelings associated with the stressor, using cognitive transformations that enable finding new avenues of solving the problem and the elicitation of positive emotions as well as positive expectations about the possibility of solving the problem. Efficiency also includes taking action to solve the problem.

Constructs similar to efficiency include positive reinterpretation and growth (Carver et al., 1989), active coping/rational coping (Lyne and Roger, 2000; Stowell et al., 2001), and proactive coping (Schwarzer, 2001). It seems that growth reflects efficiency and problem solving, whereas positive reinterpretation evinces positive emotional coping. These strategies can be considered separately, but they may also be seen as complementary ways of coping with stress. Therefore, positive reinterpretation and growth involves a combination of both problem solving and positive emotional coping.

Importantly, exploratory analysis of the COPE scales corroborated the existence of a construct similar to efficiency (Lyne and Roger, 2000; Stowell et al., 2001). Lyne and Roger (2000) and Stowell et al. (2001) identified a factor consisting of active coping, planning, suppression of competing activities, positive reinterpretation and growth, acceptance as well as restraint coping.

Another construct resembling efficiency is proactive coping, which is defined as “an effort to build up general resources that facilitate promotion toward challenging goals and personal growth” (Schwarzer, 2001, p. 406). Both efficient copers and proactive individuals are active and interpret difficulties as “eustress.” Proactive persons perceive life as “full of abundant resources” (Greenglass et al., 1999, p. 5) and efficient copers reinterpret a stressful situations in positive terms. Importantly, proactive behavior aims at life improvement and may partially solve a problem even before it emerges (Schwarzer, 2001). Proactive coping is associated with a set of COPE strategies similar to efficiency, positively correlated with active coping and planning, and inversely correlated with behavioral disengagement and self-blame (Schwarzer and Taubert, 2002).

#### Definition of Helplessness

In the CCM, helplessness is a configuration of problem avoidance and negative emotional coping. Helplessness includes not acknowledgment of the thoughts and feelings associated with the problem, using cognitive transformations, which elicit negative expectations as to the possibility of dealing with the problem as well as negative emotions (e.g., internalization the negative aspects of the problem, preoccupation with one's exaggerated limitations, and negative aspects of the situation).

Certain constructs resemble helplessness as defined above; these are: emotion-oriented coping (Endler and Parker, 1999) and maladaptive coping (Dunkley and Blankstein, 2000). Moreover, helplessness is reflected in the internalization of negative aspects of stressful situations, e.g., causes of discrimination (Wei et al., 2010) or oppression (American Psychological Association, 2007). In the feminist model of psychological practice (American Psychological Association, 2007), the internalization of persecution is a major contributor to distress among women. Moreover, members of ethnic minorities with greater internalization of discrimination score lower on self-esteem, and higher on self-blame, and behavioral disengagement (Wei et al., 2010).

One of the constructs similar to helplessness is emotion-oriented coping, defined as self-oriented efforts aimed at reducing stress through emotional responses (e.g., anger, blaming oneself for being too emotional), self-preoccupation, and fantasizing (daydreaming). These responses can increase stress (Endler and Parker, 1999). It might be presumed that helplessness and emotion-oriented coping have a similar conceptual range. Emotional responses typical of emotion-oriented coping seem akin to negative emotional coping, whereas self-preoccupation and fantasizing are related to problem avoidance. Some items (“Worry about being unable to cope,” “Focus on my inadequacies”) (Endler and Parker, 1999) directly correspond to helplessness. Moreover, emotion-oriented coping is associated with strategies involving problem avoidance and negative emotional coping (Endler and Parker, 1990; Boysan, 2012). Emotion-oriented coping is correlated with focus on and venting of emotions, avoidance (Boysan, 2012), wishful thinking as well as self-blame (Endler and Parker, 1990).

Helplessness may also be expected to exhibit correlations with some other CISS coping styles. As mentioned above, task-oriented coping is similar to problem solving, whereas distraction resembles problem avoidance. Thus, helplessness could be negatively related to task-oriented coping and positively with distraction. Maladaptive coping (high emotion-oriented, low task-oriented, high distraction) was recognized on the basis of the CISS (Dunkley and Blankstein, 2000). Emotion-oriented coping is the strongest indicator of maladaptive coping (Dunkley and Blankstein, 2000). The above interpretation of CISS styles is reflected in results from other studies (McWilliams et al., 2003; Cohan et al., 2006). Depression is associated positively with the emotion-oriented style (McWilliams et al., 2003; Cohan et al., 2006), and negatively with task-oriented coping (Cohan et al., 2006). Similarly, anxiety is positively correlated with emotion-oriented coping (McWilliams et al., 2003; Cohan et al., 2006) and distraction as well as inversely with task-oriented coping (Cohan et al., 2006).

The existence of helplessness has found some support in various analyses of dispositional Brief COPE (e.g., Carver, 1997; Knoll et al., 2005; Snell et al., 2011; Doron et al., 2014). Snell et al. (2011) found that denial, behavioral disengagement, self-blame and venting formed one factor. Doron et al. (2014) noted that denial, behavioral disengagement, self-blame and substance use loaded the same factor. Knoll et al. (2005) revealed the existence of a factor composed of denial, self-blame, and venting. It seems that the construct of helplessness is at the root of all above-mentioned categories from Brief COPE (Knoll et al., 2005; Snell et al., 2011; Doron et al., 2014).

Moreover, the interpretation of efficiency and helplessness as the opposite poles of one dimension finds confirmation in some studies (Livneh et al., 2000; Finset et al., 2002). Livneh et al. (2000) after analyzing the dispositional Brief COPE and selecting scales from the Coping Strategies Inventory (CSI, Tobin et al., 1989), found three bipolar dimensions, with the first encompassing most of the coping scales. Active coping, planning, positive reframing, and acceptance represented the positive pole of the first dimension, while substance use (from the Brief COPE), self-criticism, and social withdrawal (from the CSI) represented the opposite pole. Analogously, Finset et al. (2002), who relied on the Brief Approach/Avoidance Coping Questionnaire (BACQ), observed that approach and avoidance formed one bipolar dimension. Approach included solving the problem, seeking social support, and optimism toward the problem, whereas avoidance manifested in resignation and withdrawal (Finset et al., 2002). Bipolar dimensions from Finset et al. (2002) and Livneh et al. (2000) find reflection in the efficiency–helplessness distinction.

#### Mechanisms Underlying Efficiency and Helplessness

It may be presumed that efficiency and helplessness are associated with mindfulness in opposite ways. Moreover, it can be expected that both are related to cognitive reappraisal–expressive suppression (Gross and John, 2003) and action orientation–state orientation (Kuhl, 1981, 2000).

##### Mindfulness

Mindfulness is “the clear and single-minded awareness of what actually happens to us and in us at the successive moments of perception” (Nyanaponika, 1972, p. 5). The definitions of efficiency and helplessness refer to the awareness of one's relationship to the stressful situation vs. not acknowledging it. Efficiency and helplessness may be expected to have a positive and negative relationship with mindfulness, respectively.

Mindfulness is related to coping with stress (Palmer and Rodger, 2009; Weinstein et al., 2009) with mindful individuals being more likely to employ approach coping (i.e., active coping, acceptance, positive reinterpretation, and growth) and less likely to use avoidance coping (i.e., denial as well as behavioral and mental disengagement) (Weinstein et al., 2009). Palmer and Rodger (2009) reported positive correlations of mindfulness with rational coping (a type of problem-solving coping) and negative with emotional and avoidance coping. Mindfulness-based intervention reduces the use of disengagement coping (Cousin and Crane, 2016) and emotion-oriented coping (Messer et al., 2016).

##### Emotion regulation processes

Gross and John (2003) distinguished two emotion regulation processes: cognitive reappraisal and expressive suppression. Cognitive reappraisal is conceptualized as a “form of cognitive change that involves construing a potentially emotion-eliciting situation in a way that changes its emotional impact” (Gross and John, 2003, p. 349; cf. Lazarus and Alfert, 1964). In turn, expressive suppression is a “form of response modulation that involves inhibiting ongoing emotion-expressive behavior” (Gross and John, 2003, p. 349). Reappraisal is an antecedent-focused process, while suppression is a response-focused emotion regulation strategy. Reappraisal appears early in the emotion generation process, so that it can effectively change the entire further emotion route. In turn, suppression occurs late in the process of emotion generation. It is not useful in the reduction of negative emotion experience, but it can successfully limit the expression of negative emotions. The side effect of this strategy can be the suppression of positive emotions. By contrast, reappraisal efficiently reduces the experience and behavioral manifestation of negative emotions (Gross and John, 2003).

Cognitive reappraisal is correlated with positive reinterpretation and growth from the COPE (Gross and John, 2003; Balzarotti et al., 2010). Cognitive reappraisal and expressive suppression are associated with the problem-focused coping and avoidance, respectively (Williams and Hasking, 2010). In addition, according to Amstadter and Vernon (2008), they are related to the use of problem engagement (including problem solving and cognitive restructuring) and emotion disengagement (encompassing self-criticism and social withdrawal), respectively.

Cognitive reappraisal and mindfulness may share a common underlying mechanism, which could be decentering (Hayes-Skelton and Graham, 2013). Safran and Segal (1996, p. 117) conceptualized decentering as the ability to “step outside of one's immediate experience, thereby changing the very nature of that experience.” Both mindfulness (Gecht et al., 2014) and positive reappraisal (Fresco et al., 2007; Naragon-Gainey and DeMarree, 2017) are connected with decentering. Interestingly, suppression revealed negative association with decentering (Fresco et al., 2007). Hayes-Skelton and Graham (2013) found that decentering may be the underpinning mechanism behind both cognitive reappraisal and mindfulness. Decentering positively correlated with self-esteem (Fresco et al., 2007; Naragon-Gainey and DeMarree, 2017) and negatively with rumination (Naragon-Gainey and DeMarree, 2017). It might be presumed that decentering is connected with efficiency.

##### Action vs. state orientation

Kuhl (1981, 2000) developed the idea of action vs. state orientation. The first refers to the focus on task-relevant cognitions, while the second involves ruminating after failure. Kuhl (1981) investigated the moderating role of action vs. state orientation in the effect of learned helplessness. He proved that state-oriented individuals revealed deterioration in task performance in uncontrollable failure. In turn, people high in action orientation displayed lack of evidence of the learned helplessness effect (Kuhl, 1981). Further studies demonstrated that volitional and efficient emotion regulation is the main mechanism that differentiates action vs. state orientation (Koole and Jostmann, 2004).

Experiences of emotions are strongly linked with access to extension memory, which is conceptualized as a central executive system based on parallel processing (Kuhl, 2000). Its parallel nature enables the integration of many different self-representations, preferences, feelings, so that an individual can consciously choose a goal, optimize the ways of its implementation as well as more effectively regulate his or her emotions. Positive emotions increase and negative emotions limit the access to extension memory, which influences task performance (cf. Bolte et al., 2003). When a person high in state orientation is confronted with stress, his or her extension memory becomes dissociated from lower-level processes (Koole et al., 2005). Therefore, overarching goals and self-knowledge are detached from new experiences, which results in a worse task performance (Kuhl, 1981; Brunstein and Olbrich, 1985), rumination (Kuhl and Baumann, 2000) and a tendency to attribute others' actions to the oneself (Kuhl, 1992).

The afore-discussed distinction can be associated with the CCM, i.e., action vs. state orientation are similar to efficiency vs. helplessness, respectively. Flexibility of efficiency requires a good access to extension memory, while helplessness represents an inhibited access. Interestingly, the consequences of state orientation resemble helplessness, e.g., with regard to internalization.

In summary, the following constructs can be considered as mechanisms underlying efficiency–helplessness: mindfulness (Nyanaponika, 1972), cognitive reappraisal–expressive suppression (Gross and John, 2003), action orientation–state orientation (Kuhl, 1981, 2000).

### Preoccupation With the Problem vs. Hedonic Disengagement

Some coping strategies can be correlated with the dimensions of problem coping and emotion coping with opposite signs. For instance, problem-focused aggression and problem-focused crying (Band and Weisz, 1988) are linked to problem solving and negative emotional coping. On the other hand, a combination of problem avoidance and positive emotional coping may result in, e.g., hedonistic escapism (including humor and substance use) (Deisinger et al., 1996). A configuration of problem solving and negative emotional coping is identified as preoccupation with the problem while problem avoidance concurrent with positive emotional coping is termed hedonic disengagement.

#### Definition of Preoccupation With the Problem

Preoccupation with the problem corresponds to a combination of problem solving and negative emotional coping. An individual preoccupied with a problem exhibits a high tendency to take action to solve the problem and a low tendency to maintain his or her momentary well-being. Faced with a stressor, the person is alert, actively focuses on the information about the problem (including unpleasant facts), blocks thoughts that might be distracting (even the thoughts concerning one's own needs). The individual does not want to miss the opportunity, worrying that something bad will happen if he or she does not solve the problem right away. The individual tries to thoroughly understand the problem and makes comprehensive preparations in an effort to promptly find a solution.

Preoccupation with the problem is somewhat similar to confrontive coping (see Table 4), which is more often used by individuals perceiving a situation as a threat resulting in financial difficulties. This way of coping is employed more frequently if the people concerned believe they are capable of changing the situation (Folkman et al., 1986a). The second construct which shares some common characteristics with preoccupation with the problem is suppression of competing activities (see Table 4), which is part of problem-focused coping (Carver et al., 1989) and demonstrated weak positive relationship with higher depression severity (Kato, 2013).

Similarly, confrontive coping is correlated with distress (Folkman et al., 1986b) and negative emotions (Folkman and Lazarus, 1988). Blankstein et al. (1992) found a relationship between confrontive coping and test anxiety. Importantly, the group of coping strategies reflecting preoccupation with the problem (i.e., logical analysis and venting of unpleasant emotions) is associated with cognitive and somatic competitive state anxiety in athletes during sports contests (Gaudreau and Blondin, 2002). Athletes, who perceive their anxiety as facilitative exhibit greater use of suppression of competing activities than those who interpret it as debilitative (Ntoumanis and Biddle, 2000).

The configuration of coping constructs corresponding to preoccupation with the problem seems to be related to insight in psychosis (Cooke et al., 2007), some forms of workaholism (Andreassen et al., 2012), and perfectionism (Noble et al., 2014; Gong et al., 2015). The two components of perfectionism are perfectionistic concerns and perfectionistic strivings (Frost et al., 1993; Gotwals et al., 2012). Perfectionistic strivings are similar to preoccupation with the problem, they are positively associated with problem-focused coping (Noble et al., 2014), socioemotional coping (i.e., focus on and venting of emotions as well as seeking social support; Gong et al., 2015), and negatively with avoidant coping (Noble et al., 2014; Gong et al., 2015). Perfectionistic strivings reveal a weak but consistent relationship with depression (Smith et al., 2016). Other authors have found a relationship between perfectionistic strivings and workaholism (Clark et al., 2010; Stoeber et al., 2013).

Shimazu et al. (2010) found workaholism to be related to active coping and emotional discharge. In another study, a component of workaholism (i.e., work involvement) was positively associated with active problem solving and a depressive response pattern, and inversely with passive avoidance (Andreassen et al., 2012). Thus, one could argue that preoccupation with the problem may contain both adaptive and dysfunctional elements.

It appears reasonable that higher awareness of symptoms in psychosis could be connected to a focus on information concerning the threat or unpleasant problem. Indeed, insight factors in schizophrenia are correlated positively with suppression of competing activities, planning, seeking social support (instrumental, emotional), and negatively with mental and behavioral disengagement as well as positive reinterpretation and growth (Cooke et al., 2007). A combination of all of these strategies with the exception of social support reflects preoccupation with the problem.

#### Definition of Hedonic Disengagement

Hedonic disengagement is a combination of problem avoidance and positive emotional coping. Hedonic disengagement involves avoidance of information on the problem and a strong tendency to maintain momentary well-being. An individual uses cognitive transformations which enable give an exaggerated feeling of control over the problem (a form of internalization) and decrease its importance (problem devaluation). Hedonic disengagement also involves disregarding the problem, low engagement in solving it, postponing the task, or giving up the search for a solution.

Hedonic disengagement includes a tendency for exaggerated perception of control in stressful encounters, which has been analyzed and discussed at length by several authors (Taylor et al., 1984; Lowery et al., 1993; Zoellner and Maercker, 2006). While a self-generated feeling of control is associated with better adjustment in cancer (Taylor et al., 1984; Lowery et al., 1993; Barez et al., 2007), it may not be adaptive when the beliefs concerning the disease are not borne out by the facts (Christensen et al., 1991; Tomich and Helgeson, 2006). Exaggerated internal control is negatively correlated with depression and anxiety, but positively with mania (Berrenberg, 1987).

Moreover, the definition of hedonic disengagement resembles hedonistic escapism (Deisinger et al., 1996). Similarly, Sica et al. (1997) have identified one factor containing humor, substance use, denial as well as mental and behavioral disengagement. Interestingly, both constructs include humor, which contains at least two strategies. One of them resembles positive emotional coping, while the other represents hedonic disengagement (referring item: “I make fun of the situation” Carver et al., 1989).

Hedonic disengagement seems to share certain characteristics with the following coping constructs: seek relaxing diversions (Frydenberg and Lewis, 1993), relaxing (Patterson and McCubbin, 1987), soothing distraction (Gol and Cook, 2004). In their concept map analysis, Gol and Cook (2004) identified soothing distraction, referring to a calming and relaxing distraction. In two-dimensional space, this construct is located between self-management/relaxation (a strategy of control of emotions) and active distraction (akin to CISS distraction and COPE mental disengagement) (Gol and Cook, 2004).

Some stress responses enable detachment from the problem, e.g., social diversion. Social diversion represents stressor avoidance in conjunction with positive emotional coping (seeking out other people, e.g., talking to a friend) (Endler and Parker, 1999). Social diversion is associated with life satisfaction (Harper, 2012; Saklofske et al., 2012), positive affect (Saklofske et al., 2012), and perceived social support (Ponizovsky et al., 2004). In some studies social diversion has not been correlated with distress (Ritsner et al., 2003; Brands et al., 2014), while in others a negative relationship has been found (Ponizovsky et al., 2004; Harper, 2012). One paper has reported a weak inverse correlation for women and no effect for men (Cohan et al., 2006).

Coping responses similar to hedonic disengagement are also related to some dysfunctional tendencies (Huflejt-Łukasik and Czarnota-Bojarska, 2006; Oginska and Oginska-Bruchal, 2014). Individuals with a strong tendency for hedonic disengagement may be expected to have a low ability to adjust their behaviors (e.g., auto-presentation) to signals from their environment, i.e., they demonstrate low self-monitoring (Snyder, 1974). Indeed, social diversion reveals a negative relationship with self-monitoring (Huflejt-Łukasik and Czarnota-Bojarska, 2006). Furthermore, social diversion has been shown to be connected with susceptibility to seasonal fluctuations of mood, energy levels, and sleep length in a non-clinical sample (Oginska and Oginska-Bruchal, 2014). It seems that hedonic disengagement contains a potential for both adaptive and maladaptive aspects.

## The Prospect of Integrating Various Coping Constructs Within The CCM

Coping categories from the WCQ, COPE, and CISS can be assigned to their counterparts in the CCM, but it should be noted that the latter conceptualizes coping as a disposition. While this is consistent with the COPE and CISS, the WCQ is designed to measure situational coping. Therefore, analysis of the relationships between the CCM coping styles and the WCQ coping strategies requires some caution. The integration of CCM coping styles and categories from other coping models is given in Table 4. The CCM can accommodate six of the eight coping strategies from the model by Folkman et al. (1986a). Three categories are located between two CCM styles: confrontive coping is related to preoccupation with the problem and negative emotional coping, escape–avoidance to problem avoidance, and helplessness, and distancing to hedonic disengagement and problem avoidance. Self-controlling and seeking social support have no equivalents within the CCM. The four WCQ factors correspond to the poles of the two dimensions: problem solving, problem avoidance, preoccupation with the problem, and hedonic disengagement. The other two factors are associated with one pole of the remaining two CCM dimensions, that is, negative emotional coping and efficiency, respectively.

Ten of the 15 COPE strategies can be located within the CCM. Three of them are associated with more than one CCM coping style: suppression of competing activities resembles preoccupation with the problem and problem solving, humor corresponds to hedonic disengagement and positive emotional coping, whereas substance use to hedonic disengagement and problem avoidance. However, the location of five COPE strategies (restraint coping, seeking social support for instrumental reasons, seeking social support for emotional reasons, acceptance, turning to religion) in the CCM seems to be problematic.

Research on the COPE has revealed solutions with different numbers of factors: three (e.g., Stowell et al., 2001), four (Carver et al., 1989), or five (Deisinger et al., 1996; Sica et al., 1997). Only two factors have been replicated across all studies: avoidance (composed of denial and behavioral and mental disengagement) as well as venting of emotions/ social support (incorporating both seeking social support for instrumental and emotional reasons and focus on and venting of emotions) (Carver et al., 1989; Deisinger et al., 1996; Stowell et al., 2001). Both focus on and venting of emotions and seeking social support for emotional reasons seem to be associated with expression of negative emotions (a sample item concerning seeking social support for emotional reasons: “I talk to someone about how I feel”). As mentioned above, avoidance is similar to problem avoidance.

The results of various exploratory analyses of the COPE, which included both aforementioned factors as well as other categories, can be reconciled within the CCM. In the four-factor solution obtained by Carver et al. (1989), the first factor (consisting of active coping, planning, and suppression of competing activities) corresponds to problem solving, the second (venting of emotions/social support) to negative emotional coping, the third (avoidance) to problem avoidance, and the fourth (acceptance, restraint coping, and positive reinterpretation and growth) to efficiency. The three dimensions from the CCM find reflection in the above solution. Deisinger et al. (1996) distinguished five factors: problem-focused coping (containing active coping, planning, suppression of competing activities, restraint coping), positive reappraisal (acceptance, turning to religion, positive reinterpretation, and growth), hedonistic escapism (humor and substance use), avoidance, and venting of emotions/ social support. Problem-focused coping and avoidance correspond to both poles of the problem coping dimension. All the other factors resemble one pole from the remaining three CCM dimensions each–efficiency, hedonic disengagement, and negative emotional coping.

Sica et al. (1997) obtained a five factor-solution: the first factor (including active coping, planning, and suppression of competing activities) is similar to problem solving, the second one (humor, substance use, denial and mental, and behavioral disengagement) to hedonic disengagement, the third one (venting of emotions/ social support) to negative emotional coping, the fourth one (positive reinterpretation and growth, acceptance, and restraint coping) to efficiency, and the fifth one is turning to religion. With the exception of the last one, each factor stands for one pole of one of the four dimensions of the CCM.

The CISS coping categories seem to be fully compatible with the CCM. The task-orientation categories from the CISS resemble the problem coping dimension, i.e., task-oriented coping corresponds to problem solving and distraction is akin to problem avoidance. In turn, the CISS person-orientation constructs refer to a configuration of problem avoidance and the two poles of the emotion coping dimension, i.e., emotion-oriented coping is similar to helplessness, whereas social diversion to hedonic disengagement.

Interesting results have been reported from a study on the genetic foundations of coping styles (Kozak et al., 2005). An analysis based on genetic variances demonstrated negative correlations between task-orientation categories (i.e., task-oriented coping and distraction, *r* = −0.28). (Kozak et al., 2005). These negatively correlated CISS categories were assigned to the opposite regions of the CCM.

Basically, all CISS constructs may be expressed in terms of the CCM. The two remaining models revealed some difficulties in terms of unequivocal placement of the following categories within the CCM: two of the eight strategies proposed by Folkman et al. (1986a) and five of the 15 strategies from the model by Carver et al. (1989). Most categories from all three discussed coping models can be related to the CCM.

Finaly, it is worth noting that CCM is somewhat similar to the model of Gol and Cook (2004), especially in terms of the two basic dimensions: problem coping vs. emotion coping from the CCM and approach-avoidance vs. emotional equilibrium-disequilibrium from Gol and Cook's proposal. However, these authors identified nine clusters, two of them reflecting approach coping and seven–avoidance coping, with one cluster (passive cognitive distraction) occupying the center of the concept map (Gol and Cook, 2004). In contrast, the CCM is a full circumplex model consisting of eight evenly distributed coping styles equidistant from the center of the circle.

### Social Forms of Coping

Social coping acts may be represented in the CCM in three ways. First, social coping responses could be located in different areas of the circumplex. Second, all of the eight CCM coping styles can have non-social forms and social equivalents. Similarly, Connor-Smith et al. (2000) argued that social support may be used for many reasons and Skinner et al. (2003) stated that all individual coping responses may have social counterparts. Third, it is conceivable that an exhaustive description of coping structure would require a third dimension, namely, social-focused coping. In this case, one should consider a spherical structure of coping, similar to that of vocational interests (Tracey and Rounds, 1996). Then, the question would arise as to whether the additional dimension is bipolar, and, if so, what poles it contains.

There is yet a fourth option as, the CCM can be interpreted within different domains analogously to the interpersonal circumplex (Leary, 1957), which has been employed to describe and explain the structure of interpersonal values (Locke, 2000), interpersonal problems (Alden et al., 1990), interpersonal self-efficacy (Locke and Sadler, 2007) as well as children's social goals (Ojanen et al., 2005). Thus, the CCM could be interpreted on different levels to describe acts of social coping as well as coping with particular stressors (e.g., academic stress, illness, job stress, family stress etc.). In this case, social coping would represent one of the many applications of the circular continuum. At this stage, it would be premature to accept or reject any of these possible relations between social coping and the CCM.

## Applications of the Coping Circumplex Model

### The Potential of the CCM to Predict Mental Health

It might be presumed that efficiency and helplessness are related to hope, self-esteem, and mental health problems. Moreover, it seems that preoccupation with the problem demonstrates a relationship with the type A behavior pattern (TABP).

#### Hope and Hopelessness

Both efficiency and helplessness entail expectations about the effects of actions and certain elements of the conceptualizations of these constructs are evocative of hope and hopelessness. Snyder et al. (1991, p. 570) defined hope as “a cognitive set that is composed of a reciprocally derived sense of successful (a) agency (goal-directed determination) and (b) pathways (planning of ways to meet goals).” Hope conceptualized and operationalized in different ways is related to the use of problem-focused coping (Snyder et al., 1991; Litman, 2006; Caretta, 2011) and positive reinterpretation (Litman, 2006; Caretta, 2011). Inverse associations have been found between hope and avoidance coping (Litman, 2006; Caretta, 2011) as well as self-blame (Caretta, 2011).

It is worth looking into the relationship between coping and hopelessness. The latter denotes negative expectations about future life (Beck et al., 1974). Individuals scoring high on hopelessness prefer greater use of emotion-focused disengagement and lower use of problem-focused engagement (Taft et al., 2007). Hopelessness is positively correlated with avoidance coping and inversely with cognitive reconstruction (O'Connor and O'Connor, 2003). Hopelessness is conceptually related to other variables, such as diminishment (Litman and Lunsford, 2009).

#### Self-esteem and Diminishment

Diminishment is defined as decreased self-esteem, feelings of helplessness, and higher pessimism (Litman and Lunsford, 2009), and can be predicted from focus on and venting of emotions and behavioral disengagement (Litman and Lunsford, 2009). Self-esteem exhibits the opposite pattern of correlations with coping strategies (Scheier et al., 1994; Myers and Rosen, 1999). Individuals with higher self-esteem demonstrate less emotion-oriented coping (Leandro and Castillo, 2010; Geyh et al., 2012) and avoidance coping (Carver et al., 1989; Scheier et al., 1994; Myers and Rosen, 1999). Self-esteem is associated with positive emotional coping strategies, such as self-love and self-acceptance (Myers and Rosen, 1999), as well as positive reinterpretation and growth (Carver et al., 1989; Scheier et al., 1994). People with higher self-esteem show a greater preference for problem-focused coping (Carver et al., 1989; Scheier et al., 1994; Myers and Rosen, 1999), task-oriented coping (Leandro and Castillo, 2010; Geyh et al., 2012), and proactive coping (Umana-Taylor et al., 2008).

#### Mental Health Problems and Distress

A combination of CISS categories (i.e., high emotion-oriented coping, high distraction, and low task-oriented coping) form a maladaptive coping construct which strongly predicts distress (Dunkley and Blankstein, 2000). Maladaptive coping is thought to manifest helplessness. The above interpretation of CISS styles is reflected in other studies (e.g., Endler and Parker, 1994; Cohan et al., 2006). In conclusion, the configuration of coping styles from CISS resembling helplessness predicts mental health problems (Endler and Parker, 1994; Leandro and Castillo, 2010).

In Kato meta-analysis 2013, eight COPE coping strategies were correlated with general distress (*r* > 0.10): active coping, positive reinterpretation and growth (negatively) as well as focus on and venting of emotions, self-blame, denial, mental and behavioral disengagement, and restraint coping (positively). With the exception of restraint coping, a configuration of the remaining seven strategies reflects the efficiency–helplessness dimension. Similarly, in a meta-analysis by Li et al. (2012) dysfunctional coping (containing, e.g., avoidance, denial, emotional discharge) was correlated with proneness to anxiety and depression. It seems that the efficiency–helplessness concept provides the most parsimonious explanation of relationships between various coping constructs from different inventories and distress (e.g., Dunkley and Blankstein, 2000; Li et al., 2012; Kato, 2013).

#### TABP

Spence et al. (1987) demonstrated that TABP contains two relatively independent components: achievement striving and impatience-irritability. The former, but not the latter, has been found to be correlated with problem-focused coping and higher course grades (Lee et al., 1993), whereas impatience-irritability has been shown to be positively correlated with distress (Lee et al., 1993) and perceived stress (Day and Jreige, 2002).

While some studies have used two components of TABP, others have scrutinized correlates of the overall score. General TABP has been associated with problem-focused coping (e.g., suppression of competing activities) and focus on and venting of emotions (Carver et al., 1989), and has been consistently linked to distress (Suls and Wan, 1989; Sogaard et al., 2008) and perfectionism (Flett et al., 1994).

In conclusion, efficiency is associated with the positive aspects of mental health, i.e., hope (Snyder et al., 1991) and self-esteem (Scheier et al., 1994; Myers and Rosen, 1999), whereas helplessness is linked to the negative aspects, i.e., hopelessness (Beck et al., 1974), diminishment (Litman and Lunsford, 2009), and mental health problems (Li et al., 2012; Kato, 2013). Furthermore, a configuration of coping strategies similar to preoccupation with the problem has revealed a relationship with TABP (Carver et al., 1989).

#### Associations Between Coping and Mental Health

In previous research, mental health indicators have been found to be associated with various coping scales within individual psychometric measures. For example, self-esteem has been related to two of the three CISS coping styles (Leandro and Castillo, 2010; Geyh et al., 2012), five of the 14 COPE strategies (Carver et al., 1989; Scheier et al., 1994) as well as eight of the 21 Coping Responses categories (Myers and Rosen, 1999). Relationships between coping and this simple unidimensional construct of mental health do not seem clear. Similarly, other variables associated with mental health (e.g., distress) have been related to a configuration of various coping constructs (McWilliams et al., 2003; Cohan et al., 2006; Kato, 2013). Moreover, these combinations of coping categories predicting mental health are not founded on a theoretical background.

The CCM offers the possibility to easily and clearly link external variables with coping. Self-esteem is associated with efficiency, whereas distress is correlated with helplessness. The CCM enables a theoretically coherent and more parsimonious explanation of mental health indicators than other coping models.

### The Prospect of Integrating Selected Emotion Regulation Strategies Within the CCM

The CCM can be related to emotion regulation strategies from Gross (Gross and John, 2003) as well as Wells and Davies (1994). Wells and Davies (1994) identified five dimensions of control of unpleasant and undesirable thoughts: distraction (sample item: “I do something that I enjoy”), social control (e.g., “I ask my friends if they have similar thoughts”), worry (e.g., “I focus on different negative thoughts”), punishment (e.g., “I punish myself for thinking the thought”), and reappraisal (e.g., “I try to reinterpret the thought”). Distraction is linked to hedonic disengagement and problem avoidance, worry to negative emotional coping, punishment to helplessness and negative emotional coping, while reappraisal to problem solving. Social control has no counterpart within the CCM coping styles. With the exception of social control, each strategy of thought control is associated with one of the four dimensions of the CCM.

As already mentioned, also cognitive reappraisal and expressive suppression (Gross and John, 2003) can be located within the CCM. Cognitive reappraisal and expressive suppression exhibit similarities to efficiency and helplessness, respectively. Presumably, various emotion regulation strategies might be expressed in terms of the CCM.

### Correlates of Posttraumatic Growth

The CCM can be applied to interpret posttraumatic growth (PTG), which consists of positive changes resulting from coping with trauma (Tedeschi and Calhoun, 1996). According to Zoellner and Maercker (2006), a comprehensive description of predictors of PTG requires two components: functional, self-transcending, or constructive and illusory, self-deceptive, or dysfunctional. The first component concerns recreating an understanding of the individual's beliefs about the world (Batten and Oltjenbruns, 1999; Tedeschi et al., 2007), spiritual development (Calhoun et al., 2000), problem-focused coping, positive reinterpretation, and acceptance (Linley and Joseph, 2004). The second component is related to some forms of self-deception (e.g., exaggerated perception of control, unrealistic optimism), and it can predict good adjustment to stressful events (Taylor and Armor, 1996) as well as lower cardiovascular reactivity (Why and Huang, 2011). The model of the two components of PTG has been empirically verified (Zoellner et al., 2008).

Various combinations of these two constructs are possible: “if the illusory perception of PTG co-exists with deliberate thinking about the trauma and does not preclude active coping efforts, then, it may serve as a short-term adaptive palliative coping strategy” (Zoellner and Maercker, 2006, p. 640). The possibility of the existence of different configurations of the two components is convergent with the circumplex structure of coping. It can be presumed that the functional component is associated with problem solving and efficiency, whereas the illusory component refers to positive emotional coping and hedonic disengagement.

### Psychological Interventions and the CCM

As it has been noted, to date coping models have not provided a sound theoretical basis for psychological interventions and there is a gap between coping theory and clinical practice (Coyne and Racioppo, 2000). Fortunately, there is also an increasing body of research concerning the effectiveness of different interventions (Foa et al., 1999; Steenkamp et al., 2015). According to a meta-analysis of PTSD psychotherapies for war veterans, the two most effective methods are cognitive therapy and exposure therapy (Steenkamp et al., 2015). The idea of cognitive therapy is consistent with the coping structure postulated by the CCM, especially in the dimensions of efficiency and helplessness. For example, cognitive biases in depressed individuals and trauma victims are similar to the cognitive responses included in helplessness as a coping style. According to Beck (Beck and Dozois, 2011), depression is associated with negative thoughts about oneself, one's experiences, and the future. Analogously, trauma victims often demonstrate inappropriate guilt related to their experiences, e.g., a veteran might blame himself for the death of his friend. These biases result in negative emotions and impaired functioning (Foa et al., 1999).

Cognitive training raises awareness of the content of one's thinking and beliefs, enabling intentional modification of unrealistic cognitive schema, and automatic thoughts to improve emotional and behavioral functioning (Beck and Dozois, 2011). However, interventions do not rely on a simple replacement of negative representations with positive thoughts. During cognitive therapy, individuals shift their appraisals from unhealthy thoughts to more evidence-based ones (Beck and Dozois, 2011). This refers to the idea of decentering, which is regarded as a useful therapeutic mechanism (cf. Safran and Segal, 1996) associated with efficiency. Changes of cognitive processes in cognitive therapy can be interpreted as a transition from helplessness to efficiency (cf. Leddy et al., 2013). Cognitive therapy should increase awareness of one's own cognitive processes and reduce cognitive distortions eliciting negative emotions and negative expectations as to one's own competence (e.g., filtering, catastrophizing, personalization). Simultaneously, therapy improves the ability to use cognitive modifications to find new ways of problem solving (problem-solving techniques). It also stimulates the cognitive processes of elicitation of positive emotions as well as positive expectations about one' own ability to overcome problems by cognitive restructuring.

The other widely applied therapeutic method in trauma victims is exposure therapy (Steenkamp et al., 2015), which encompasses psychoeducation, exposure to trauma-related stimuli through imagination or narratives, *in vivo* exposure (exposure in natural conditions) and modification of beliefs associated with the difficult experience (Steenkamp et al., 2015). Is seems that the processes activated during exposure resemble preoccupation with the problem, which can facilitate habituation to the distress-generating stimuli.

Another method useful in stressful situations is distraction (Malloy and Milling, 2010). A particularly interesting form of this technique is virtual reality distraction, which has been reported to be effective in pain reduction (Malloy and Milling, 2010). The treated individual wears a helmet rendering a computer-simulated 3-D reality. For instance, during a painful medical procedure a child may play a game in a virtual ice-cream factory (Chan et al., 2007). Coping through virtual reality distraction is similar to CCM hedonic disengagement. Indeed, it appears that a variety of psychological interventions can be associated with the coping styles included in the CCM.

Furthermore, the CCM can be related to other techniques improving coping with stress, e.g., expressive writing. A study by Low et al. (2008) investigated three types of expressive writing: evaluating the appropriateness of one's emotional response, attending to one's emotions in an accepting way, and describing the objective details of the experience. Evaluating one's emotions reflects negative emotional coping/ helplessness, accepting one's emotional responses is similar to positive emotional coping/ efficiency, whereas a focus on objective elements of the situation resembles problem solving. If in fact different strategies can be identified within one coping mode, this could facilitate the refinement of psychological interventions and the aggregation of results from studies using diverse inventories.

### The Connection Between Appraisal and the Preferred Coping Strategy

Lazarus and Folkman (1984) argued that coping cannot be regarded as effective or ineffective independently of the situation in which it is employed. This line of reasoning is reflected in the idea of “goodness of fit” referring to a match between appraisal and the endorsed coping strategy. A greater preference for problem-focused strategies in controllable situations and a more extensive use of emotion-focused efforts in uncontrollable situations should be associated with better adjustment (Lazarus and Folkman, 1984).

Two dimensions seem to be particularly important from the point of view of the fit between appraisal and coping: efficiency vs. helplessness and preoccupation with the problem vs. hedonic disengagement. In relatively controllable situations efficiency can be deemed beneficial (Knoll et al., 2005), but also when perceived control is low, it is still associated with better outcomes (Taylor et al., 2008; Kvillemo and Bränström, 2014) or at least it is uncorrelated with distress (Ben-Zur and Zeidner, 1995). Ben-Zur and Zeidner (1995) observed that situational active coping, planning, and positive reinterpretation were uncorrelated with state anxiety and bodily symptoms during war, but were negatively linked to distress after war.

Higher scores on the active coping factor (encompassing problem-focused coping, positive reinterpretation and growth as well as seeking social support) predicted better mental health in patients with end-stage lung disease awaiting lung transplantation (Taylor et al., 2008). A configuration of situational coping strategies reflecting efficiency was associated with higher positive affect in patients undergoing cataract surgery on the day of admission and on the day of surgery (i.e., active coping, focus on positive; Knoll et al., 2005). It seems that efficiency is beneficial both in an uncontrollable condition of severe stress (i.e., awaiting lung transplantation) and a somehow more controllable situation of mild stress (i.e., cataract surgery).

The opposite of efficiency is helplessness, which can be harmful regardless of situation controllability (Fournier et al., 2002; Knoll et al., 2005; Taylor et al., 2008). Patients awaiting lung transplantation who relied on disengagement coping (including avoidance strategies as well as focus on and venting of emotions) reported poorer mental health (Taylor et al., 2008). Knoll et al. (2005) found that in patients undergoing cataract surgery, at three out of four analyzed time points, negative affect was associated with situational evasive coping (contained self-blame, denial, and venting). Fournier et al. (2002) investigated the effects of coping on the functioning of patients with different levels of perceived control. People with diabetes represented the highest control perception, rheumatoid arthritis–moderate, and sclerosis multiplex–the lowest. The authors found that situational emotion-oriented coping predicted greater distress for all patient groups (Fournier et al., 2002).

A second dimension useful in elucidating the relationship between coping and situation is preoccupation with the problem vs. hedonic disengagement. It seems plausible that under controllable conditions the former may be associated with experiencing negative emotions in the short term, but over a long term perspective it should be related to better adjustment. A configuration of problem solving (i.e., problem-focused coping, Osowiecki and Compas, 1998; Park et al., 2001; problem-management coping, Terry and Hynes, 1998) and negative emotional coping (i.e., venting, Ben-Zur and Zeidner, 1995; accepting responsibility, Penley et al., 2002) indicates preoccupation with the problem. The problem solving component of that construct seems to be adaptive when control is high and potentially harmful in uncontrollable conditions. When perceived control is high vs. low, the endorsement of problem-focused coping is correlated with better mental health (Osowiecki and Compas, 1998; Park et al., 2001). In contrast, in uncontrollable situations problem-management coping predicts increased distress (Terry and Hynes, 1998).

Presumably, the negative emotional component of preoccupation with the problem is harmful or more harmful under low control. Situational accepting responsibility predicted poorer health for uncontrollable stressors, but no correlation was found for controllable situations (Penley et al., 2002). Situational venting revealed a stronger positive correlation with distress during war than after war (Ben-Zur and Zeidner, 1995). In conclusion, preoccupation with the problem can be generally beneficial when control is high (Osowiecki and Compas, 1998; Park et al., 2001), but maladaptive when the conditions are uncontrollable (Ben-Zur and Zeidner, 1995; Penley et al., 2002).

In contrast, in a controllable situation hedonic disengagement can be moderately positively associated with distress or uncorrelated with it. For high control conditions, strategies similar to hedonic disengagement/problem avoidance (i.e., distancing, Penley et al., 2002) predict worse health. It has been found that when control is high responses similar to positive emotional coping/hedonic disengagement (i.e., humor, Eisengart et al., 2003) are unrelated to adjustment. On the other hand, in low controllable situations constructs resembling hedonic disengagement/positive emotional coping (i.e., humor, Carver et al., 1993; Eisengart et al., 2003; problem-appraisal coping, Terry and Hynes, 1998) or hedonic disengagement (i.e., humor and denial, Ben-Zur and Zeidner, 1995) are correlated with better mental health or lower distress. The construct of hedonic disengagement could provide a framework for identifying adaptable avoidance responses in uncontrollable situations.

In conclusion, when perceived control is high, the endorsement of efficiency seems to be associated with the strongest benefits and the absence of costs. In the same situations, preoccupation with the problem can be functional, but some costs are possible (e.g., transient negative emotions). In a highly controllable environment helplessness is strongly linked to distress, while hedonic disengagement is unrelated or moderately positively correlated with distress. In uncontrollable conditions, efficiency and hedonic disengagement are probably most adaptable. Under low control, helplessness and preoccupation with the problem may be harmful. Generally, efficiency is useful in the widest spectrum of situations, while helplessness is maladaptive regardless of the situation. It seems that CCM shed new light on the fit between the endorsed coping strategy and situational controllability.

## Limitations and Conclusions

This paper presents a theoretical model which empirical verification is needed. While the most significant limitation of the CCM is that it does not account for social support seeking, it is hoped to overcome some of the major problems in coping structure and stress psychology. First, the CCM supplements the set of coping categories (i.e., process, strategy, style) with coping mode. Second, it provides a platform for the synthesis of various coping constructs and creates a common denominator for the diffuse research efforts devoted to coping. Third, it may elucidate relationships between coping and some external constructs (e.g., mental health, emotion regulation) as well as enable the prediction of adjustment after trauma. Fourth, the CCM provides a linkage between coping theory and mechanisms of improvement in psychological interventions (such as cognitive therapy). Fifth, it clarifies relationships between the effectiveness of coping strategies and situation controllability. Last but not least, the CCM may foster the generation of hypotheses integrating the efforts of stress psychology and other disciplines, including clinical and emotion research.

## Author Contributions

The author confirms being the sole contributor of this work and has approved it for publication.

### Conflict of Interest Statement

The author declares that the research was conducted in the absence of any commercial or financial relationships that could be construed as a potential conflict of interest.
